# Whole genome sequencing of *Lacticaseibacillus casei* KACC92338 strain with strong antioxidant activity, reveals genes and gene clusters of probiotic and antimicrobial potential

**DOI:** 10.3389/fmicb.2024.1458221

**Published:** 2024-09-26

**Authors:** Sujatha Kandasamy, Kil-Ho Lee, Jayeon Yoo, Jeonghee Yun, Han Byul Kang, Ji Eun Kim, Mi-Hwa Oh, Jun-Sang Ham

**Affiliations:** Animal Products Research and Development Division, National Institute of Animal Science, Rural Development Administration, Wanju-gun, Republic of Korea

**Keywords:** *Lacticaseibacillus*, antioxidant, probiotics, whole genome sequencing, bacteriocin, stress-related proteins, mobile genetic elements

## Abstract

*Lacticaseibacillus casei* KACC92338 was originally isolated from Korean raw milk. The antioxidant activities and protective effect *in vitro* of this strain were evaluated extensively. The results showed that KACC92338 can tolerate hydrogen peroxide up to 2 mM and cell-free supernatant (CFS) had higher scavenging rates for DPPH, hydroxyl radical, reducing power, and iron chelating activities with 95.61 ± 1.59%, 34.10 ± 1.93%, 2.220 ± 0.82 and 81.06 ± 1.06%, respectively. Meanwhile, the CFS showed a protective effect on yeast cells against 10 mM hydrogen peroxide with a survival rate of 76.05 ± 5.65%. To explore the probiotic potential of KACC92338, whole genome assembly and gene clusters with probiotic properties were further analyzed. The genome size was 3,050,901 bp with a 47.96% GC ratio, and 63 contigs. The genome contains 3,048 genes composed of 2,981 coding sequences and 67 RNAs (including 57 tRNAs +9 rRNAs +1 tmRNA). Average Nucleotide Identity and genome-based taxonomy showed that the KACC92338 genome had close similarity with *L. casei* strains with 96% ANI. Functional annotation by EggNOG and KEGG revealed the presence of numerous genes putatively involved in carbohydrate- and amino acid-transport and metabolism, genetic information processing, and signaling and cellular processes. Additionally, several genes conferring probiotic characteristics such as tolerance to stress, heat, cold, acid, bile salts, oxidative stress, immunomodulation, and adhesion to intestinal epithelium were identified. Notably absent were acquired antibiotic resistance genes, virulence, and pathogenic factors, that prove KACC92338 is a safe strain. Besides, the defense mechanisms of KACC92338 include six prophage regions and three clustered regularly interspaced short palindromic repeat (CRISPR) arrays as acquired immune systems against mobile elements. Further, the BAGEL4 database determined antimicrobial bacteriocin clusters of class IIb: sakacin-P, Enterolysin_A, sactipeptides, and Enterocin X, which suggests the strain could exhibit a wide range of antimicrobial functions. Together, these findings show that the *L. casei* KACC92338 strain can be a potential probiotic candidate in producing functional fermented foods-, health care- and skin care products- with antioxidant properties. However, a few more mechanistic studies are necessary on the safety assurance and potential application of the strain as a probiotic agent.

## 1 Introduction

Oxidative stress refers to an imbalance between the over production of reactive oxygen species (ROS) and reduction of antioxidant defense mechanisms (Feng and Wang, 2020). Consequently, it is pivotal to protect the human body from attack of ROS, which is related to the onset and or progression of several diseases (i.e., atherosclerosis, chronic diseases, cardiovascular diseases, metabolic disorders, and neurogenerative diseases) (Valko et al., 2007; Zhou et al., 2008; Sharifpanah and Sauer, 2016). Supplementation of antioxidants have become an important means of controlling the oxidative stress. Particularly, natural antioxidants are now more in line with demand. In recent years, lactic acid bacteria (LAB) with antioxidant function have been gaining attention as an effective natural antioxidant because of their potential to alleviate the ROS accumulation in the host (Feng and Wang, 2020; Tian et al., 2022). To develop and commercialize such antioxidant LAB as supplements, they would also have to exhibit probiotic properties. A great quantity of researches reported that probiotic LAB such as *L. plantarum* KCTC 3099, *L. fermentum* ME-3, and *L. brevis* DSMZ 23034 (Lee et al., 2005; Mikelsaar and Zilmer, 2009; Amaretti et al., 2013; Mishra et al., 2015) have positive antioxidant effects in body or cells.

LAB strains have been extensively studied as probiotics because of their desired properties like consumer safety, stability, persistence in the gastrointestinal tract and antioxidation. Traditionally, the selection of probiotic strains relies on studies of their physiological (e.g., acid tolerance) and biochemical properties (e.g., enzyme production) to ensure their safety and potential health benefits. With current advances in next-generation sequencing technologies and platform, the approach has shifted to study the molecular mechanisms and genetic characteristics which can provide more precise confirmation of the taxonomy, lifestyle, and industrial potential of novel LAB strains from their genomes (Guinane et al., 2016). Combining *in silico* analysis of whole genome sequencing and genome mining tools can provide insights into the complete genetic information and presence of genes related to probiotic properties, metabolic capacities, virulence factors, antibiotic resistance genes, and hazardous metabolites (Guinane et al., 2016; Kandasamy et al., 2022), as well assuring the safety of each probiotic strain at the genome level (Toh et al., 2013; Kang et al., 2017; Chokesajjawatee et al., 2020). However, it is important to note that the genomic information cannot guarantee but only identify the key molecular players involved in the functional role/ mechanisms of the strains (Guinane et al., 2016). In accordance, (Chokesajjawatee et al., 2020) stated that the presence of macrolide (*msrA*) and beta lactamase (*penP*) resistance genes in *L. plantarum* BCC 9546 genome did not confer resistance to erythromycin and ampicillin, respectively. Similarly, identification of hemolysin III protein, the product of hly gene, is another example. Therefore, the combination of characteristic experiments and genetic information of candidate strains can turn out to be very effective to promote the evaluation system of probiotics.

*Lacticaseibacillus casei* (previously *Lactobacillus casei*) is a facultative heterofermentative LAB with significant research interest due to its industrial value and potential health benefits. *L. casei* strains has been extensively used worldwide for manufacturing milk products and other commercial purposes (Hill et al., 2018; Zheng et al., 2020). *L. casei*-01 strain has traditionally been employed as a probiotic in dairy products. *L. casei*-01 has recently been incorporated into whey-protein isolate or polysaccharide-based edible films used in meat and bakery products or fruits (Odila Pereira et al., 2018; Dianin et al., 2019; Pruksarojanakul et al., 2020). Although several functionalities of *L. casei* have been described, to date only few reports have been published describing antioxidative effects and only one have integrated functional genomic data with properties of *L. casei* (Kim et al., 2022).

To date, there is not a single *L. casei* strain from raw milk reported for functional genomic data with antioxidant properties. Therefore, it is critically important to build a comprehensive profile of the characteristics and genomic data of the newly isolated *L. casei* strain. Here, we report the antioxidant properties, whole-genome sequence, and *in silico* analysis that unravel the probiotic and technological traits of *Lacticaseibacillus casei* KACC92338 strain, isolated from raw milk samples collected from Republic of Korea (ROK) dairy farms.

## 2 Materials and methods

### 2.1 Strain information

Lactic acid bacteria (LAB) strain used in this study were originally isolated from raw milk collected from dairy farm in the ROK. Serial decimal dilutions were prepared from the raw milk and plated on MRS agar (Difco, USA) at 37°C for 2 days in an anaerobic atmosphere. The single colony was subjected to colony purification twice and identification was primarily based on cell morphology, gram staining, and catalase reactions. The cryostocks of the strain was prepared in MRS broth with 20% (v/v) glycerol and stored at −80°C.

### 2.2 Screening for resistance to hydrogen peroxide

The resistance of LAB strain to H_2_O_2_ was determined as described by Li et al. (2012), with some modifications. The overnight grown strain was inoculated at 2% (v/v) into MRS broth (control) and MRS broth supplemented with 2 mM H_2_O_2_ (sample). Every 1 h, the plate was shaken briefly and the OD600 was measured by a microplate reader (TECAN Infinite M200 Pro, San Jose, USA).


$$\mathrm{Survival rate}\;\left(\%\right)=\left(A_{\mathrm{sample}}/A_{\mathrm{control}}\right)\times 100$$

where A_sample_ and A_control_ are optical absorbance of sample and control at 600 nm, respectively.

### 2.3 Preparation of cell-free supernatant

The strain KACC 92338 was cultured in MRS broth for 24 h at 37°C and the supernatant was collected by centrifugation at 6000 g, 20 min, 4°C. The supernatant was filter sterilized using a 0.22 μm filter membrane (Millipore, MA, USA) and the resulting cell-free supernatant (CFS) was used in for antioxidant assays.

### 2.4 *In vitro* antioxidant activity

#### 2.4.1 Scavenging of DPPH free radical

The 1,1-diphenyl-2-picrylhydrazyl (DPPH) free radical-scavenging capacity of KACC 92338 was determined according to the method described by Kao and Chen (2006), with some modifications. Briefly, 1.0 mL CFS was added to same volume of 0.2 mmol/L methanol solution of DPPH. The mixture was mixed vigorously and kept in the dark for 30 min. Then absorbance was measured at 517 nm and scavenging ability is calculated as:


$$\mathrm{Scavenging activity}\;\left(\%\right)=[1-(A_{s}–A_{b}/A_{c}]\times 100$$

where A_s_ is the absorbance of sample (CFS + DPPH), A_b_ is absorbance of blank (methanol + DPPH), and A_c_ is absorbance of control (deionized water + DPPH).

#### 2.4.2 Scavenging of hydroxyl radical

The hydroxyl radical scavenging assay was conducted as described by Li et al. (2013) with slight modifications. In brief, 0.5 mL CFS was mixed into a reaction mixture containing 1.0 mL of PBS (20 mM, pH 7.4), 0.5 mL of 1,10-phenanthroline (2.5 mM, Sigma-Aldrich) and 0.5 mL of FeSO4 (2.5 mM). Then 0.5 mL H_2_O_2_ (2.5 mM) was added to activate the reaction and kept in waterbath at 37°C for 90 min. The absorbance was measured at 536 nm and free radical scavenging activity is expressed as:


$$\mathrm{Scavenging activity}\;\left(\%\right)=\left[\left(A_{s}–A_{c}\right)/\left(A_{b}–A_{c}\right)\right]\times 100$$

where A_s_ is the absorbance in presence of sample (CFS), A_c_ is the absorbance without sample (deionized water replaced CFS), and A_b_ is the absorbance of blank (deionized water replaced CFS and H_2_O_2_).

#### 2.4.3 Reducing power assay

The reducing power was determined according to the method of Oyaizu (1986) with few modifications. Briefly, CFS (500 μL) was mixed with an equal volume of each 0.2 M sodium phosphate buffer (pH 6.6) and 1% potassium ferricyanide, and the mixture was incubated at 50°C for 20 min. After cooling rapidly, 10% trichloroacetic acid (0.5 mL) was added and the mixture was centrifuged at 3000 g for 10 min. The obtained supernatant was mixed with an equal volume of deionized water and 0.1% ferric chloride solution (200 μL). The absorbance was read at 700 nm after 10 min.

#### 2.4.4 Fe^2+^-chelating assay

The Fe2 + −chelating ability was estimated using the method of Lin and Yen (1999). In brief, 0.1 mL sample was added with 0.05 mL of 2 mM FeCl2, 1.85 mL of deionized water, and 0.1 mL ferrozine (5 mM), mixed vigorously and then incubated for 10 min. The absorbance was read at 562 nm and the Fe2 + −chelating ability is calculated as:


$${Fe}^{2+}-\mathrm{activity}\;\left(\%\right)=[1-(A_{s/}A_{c}]\times 100$$

where A_s_ and A_c_ are absorbance of sample and control (deionized water replaced sample), respectively.

### 2.5 Protective effects against hydrogen peroxide toxicity on *Saccharomyces cerevisiae*

To measure the protective effect of CFS against oxidative toxicity in eukaryotic cells, *Saccharomyces cerevisiae* belonged to our lab was incubated with Glucose Peptone Yeast Extract Broth (GPY) at 28°C for 48 h with shaking at 180 rpm. The *S. cerevisiae* (0.05 mL) was inoculated into 0.95 mL of CFS and incubated for 1 h at 30°C with shaking at 180 rpm. Then H_2_O_2_ was added to the suspension at a final concentration 10 mM. After 1 h of incubation at 28°C with shaking, serial dilutions were immediately made and the surviving yeast cells were counted with Potato dextrose agar at 30°C for 48 h. Survival rate was calculated as the ratio of CFU of surviving cells to the CFU without hydrogen peroxide.

All experimental data in this study were obtained from at least three replicates and are expressed as the mean ± standard deviation (SD).

### 2.6 Whole-genome sequencing, assembly and annotation

The whole genome sequencing of KACC92338 was performed at TK Biotech and science (Jeonbuk, Republic of Korea) as mentioned in our previous study (Kandasamy et al., 2022), In brief, the genomic DNA from KACC92338 strain (grown in MRS broth aerobically at 37°C, 16 h) was extracted using Exgene™ Cell SV Kit (Cambio, Reading, UK) and its DNA concentration and purity was measured using the Quant-iT™ BR assay Kit (Invitrogen, Waltham, MA, USA). A standard genomic Illumina 350 bp paired-end library was constructed and sequenced using Illumina Novaseq 6,000 platform. The resulting sequence reads were uploaded in Galaxy^1^, and removal of adapter and low-quality reads was done using the Trimmomatic tool v0.38.1^2^. Then *de novo* assembly was carried out with Shovill v1.1.0 under default parameters by excluding contigs <100 bp. Finally, the quality of assembled sequence was assessed through the Quast v5.2.0 tool.

The presence /absence of plasmid was determined through Plasmid Finder 2.0 (Carattoli et al., 2014). A graphical genome map of the strain was visualized in the Proksee server (Grant et al., 2023). 2.2. Genome-based identification and taxonomy analysis.

Species identification of KACC92338 was determined via calculation of the Average Nucleotide Identity (ANI) and tetra indices using the JSpecies web server tool with default parameters (Richter et al., 2016). In addition, genome-based taxonomical affiliation at the species level was illustrated through a bootstrapped phylogenetic tree constructed in the Type (Strain) Genome Server (TYGS) server (Meier-Kolthoff and Göker, 2019).

### 2.7 Gene prediction and functional annotation

The gene prediction and annotation were performed using the Prokaryotic Genome Annotation System (Prokka) pipeline tool, v1.14.6 in Galaxy (Seemann, 2014) and the Rapid Annotations using Subsystems Technology (RAST) webserver (Aziz et al., 2008) with default parameters. The cluster of orthologous groups (COG) for the protein-coding genes was obtained using Egg-NOG mapper version (2.1.12) tool (Cantalapiedra et al., 2021) from the online Egg-NOG database (version 5.0). Further, a complementary functional analysis was performed using the Kyoto Encyclopedia of Genes and Genomes (KEGG) mapper/BLASTKOALA tool (Kanehisa et al., 2016). Prediction of Carbohydrate-active enzymes (CAZymes) families within the genome was annotated using the dbCAN3 meta server (Huang et al., 2018).

### 2.8 Genome mining for probiotic potential and bacteriocins

Screening of the annotated genes putative for important probiotic properties was manually predicted through Prokka, RAST, and KEGG–derived annotations, based on previous studies. The bacteriocins in the KACC92338 genome were identified using the BAGEL 4 online server^3^ (Van Heel et al., 2018).

### 2.9 Genes related to safety aspects and defense mechanism

The presence of antibiotic resistance genes in the genome was analyzed using three databases, i.e., ResFinder tool v.4.1. of the Center for Genomic Epidemiology (Bortolaia et al., 2020), Resistance Gene Identifier (RGI) tool in Comprehensive Antibiotic Resistance Database (CARD) (Alcock et al., 2020) and BlastKOALA tool in KEGG database (Kanehisa et al., 2016).

Putative virulence factors and toxin genes were searched using the Virulence finder v.2.0.3 (Joensen et al., 2014) and Virulence Factor of Bacterial Pathogen database (VFDB) (Liu et al., 2022). In addition, the BlastKOALA tool in KEGG database (Kanehisa et al., 2016) was used for inspection of virulence factors and undesirable genes. The MobileElementFinder (version 1.03) was used to detect mobile genetic elements and their relation to antimicrobial resistance genes and virulence factors (Johansson et al., 2021).

Bacterial insertion sequences were identified using ISfinder with an E-value threshold of 1e−5 (Siguier et al., 2006). The genomic plasticity analysis was performed in Island Viewer 4 platform to determine the presence of genes related to virulence factors and antibiotic resistance genes (Bertelli et al., 2017). The presence of Clustered Regularly Interspaced Short Palindromic Repeats (CRISPR) and CRISPR-associated (Cas) genes were identified using the CRISPRCasFinder (Couvin et al., 2018). Prophage regions within the genome were detected using the PHAge Search Tool Enhanced Release (PHASTER) webserver (Arndt et al., 2016).

## 3 Results and discussion

### 3.1 Resistance to H_2_O_2_

Hydrogen peroxide is a weak oxidant than hydroxyl, but can easily penetrate the cell membrane and form more active ROS and hydroxyl radicals, causing oxidative damage to DNA, proteins, and lipids (Mishra et al., 2015). The survival rate of KACC92338 strain at various concentrations of H_2_O_2_ is presented in Table 1. This result indicated that strains had a considerable tolerance toward H_2_O_2_. The addition of H_2_O_2_ hindered the growth of the strain and duration of the lag phase was prolonged with increase in H_2_O_2_ concentration (data not shown), implying that the presence of H_2_O_2_ caused oxidative damage in bacterial cells. The observations were similar to growth pattern reported earlier with *L. plantarum* MA2 (Tang et al., 2017). It could be observed that KACC92338 strain could survive the challenges until 2 mM H_2_O_2_, with a survival rate of 95.99% within 48 h, higher than that reported elsewhere (Vijayakumar et al., 2015; Mu et al., 2018). The higher survival rate might be caused by the extracellular polysaccharides produced by the bacteria (Li et al., 2023). Therefore, the cell free supernatant (CFS) of the strain was further used to explore its *in-vitro* antioxidant potential.

**Table 1:** Survival of *L. casei* KACC92338 under the stress of different concentrations of H_2_O_2_.

H_2_O_2_ conc (mM)	OD 600 nm*
Control	2.57 ± 0.11
0.5	2.35 ± 0.14
1.0	1.52 ± 0.06
2.0	0.76 ± 0.10

* Values are mean ± SD of three replicates.

### 3.2 Antioxidant properties

Several methods have been developed to evaluate the antioxidant potential of LAB strains (Gulcin, 2020). In the present study, four indices (DPPH radical, hydroxyl radical, reducing power, and Fe-chelating ability) were chosen to evaluate the antioxidant activity of KACC92338 *in vitro* in order to gather more complete information for a better understanding of the topic (Table 2). The CFS exhibited stronger scavenging activity for DPPH (95.61 ± 1.59%), reducing power (2.220 ± 0.82), hydroxyl radical (81.06 ± 1.06%) and Fe-chelating ability (34.10 ± 1.93%). The DPPH and hydroxyl radical scavenging activity of this study was much higher than to previous reports by *L. plantarum* MA2 (40.42 ± 2.19%) (Tang et al., 2017) and *L. plantarum* KM1 (Tian et al., 2022). Similarly, the iron chelating activity (81.06 ± 1.06%) of KACC92338 was much higher than reported elsewhere. The reducing power which serve as a significant indicator for potential antioxidant capacity was also seems to be remarkable for our strain. These results conclude that the CFS of KACC92338 possess good antioxidant capacity.

**Table 2:** Antioxidant activity of cell-free supernatant from *L. casei* KACC92338 determined using different assays.

Antioxidant assays	Scavenging capacities (%)*
DPPH radical	95.61 ± 1.59
Hydroxyl radical	34.10 ± 1.93
Reducing power (Abs 700 nm)	2.220 ± 0.82
Fe chelating	81.06 ± 1.06

* Values are mean ± SD of three replicates.

### 3.3 Protective effect against H_2_O_2_ toxicity on *Saccharomyces cerevisiae*

**Figure 1:**
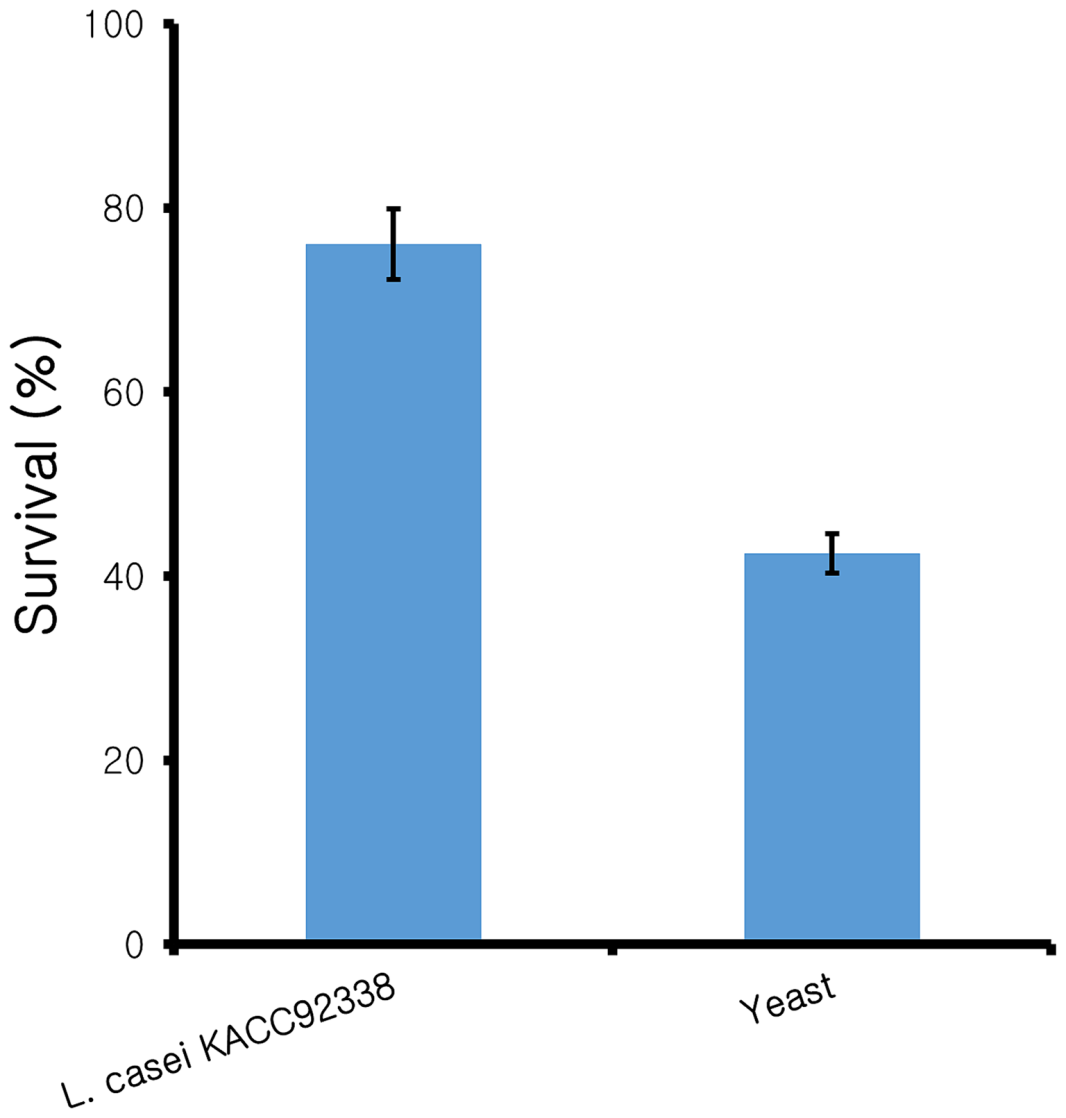
Protective effect of cell-free supernatant from *L. casei* KACC92338 on yeast cells from H_2_O_2_ oxidative damage. Values are mean ± SD of three replicates.

Since *L. casei* KACC92338 has good antioxidant capacity *in-vitro*, the protective effect of CFS against the H_2_O_2_ toxicity was determined using *S. cerevisiae.* Yeast cells was chosen as it was the simplest eukaryote and closest to the human body cells. Supplementation of antioxidants can effectively reduce the H_2_O_2_ damage to DNA, by directly reacting with H_2_O_2_ and the intermediate products of H_2_O_2_ or inhibit the peroxidase in conjunction with H_2_O_2_ can reduce the damage to cells (Tang et al., 2017). The results (Figure 1) showed that compared to control (42 ± 3.26%), cells treated with CFS showed higher viability (76.05 ± 5.65%), indicating the strongest protective effect against H_2_O_2_. These results suggested that KACC92338 is an effective scavenger for free radicals and protective agents that could help to reduce damages in human body caused by oxidative stress. Previous reports have shown that CFS supernatants from LAB are rich in antioxidant elements such as bioactive peptides, antioxidant enzymes, lipoteichoic acid or extracellular polysaccharides, carotenoids, histamine and magnesium ions (Kim et al., 2021). Therefore, whether KACC92338 can produce antioxidant molecules to improve its antioxidant performance needs to be further verified.

In conclusion, KACC92338 exhibits great *in-vitro* antioxidant activity, and signifies promising antioxidant potentials for therapeutic benefits to human health. Hence, to further understand its genetic characteristics and evaluate its probiotic potential of KACC92338 at genetic levels, whole-genome sequencing was performed.

### 3.4 General genome features

**Figure 2:**
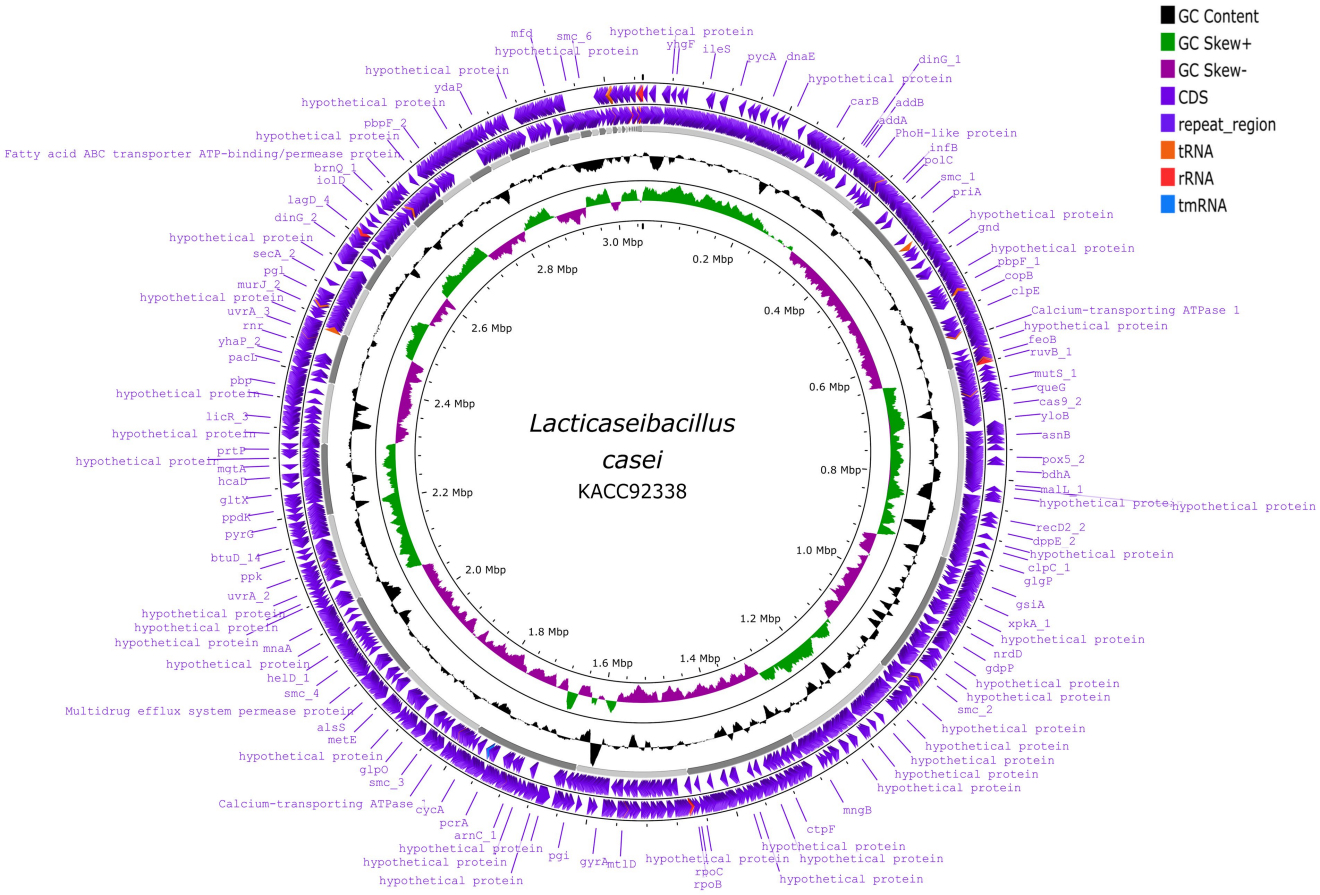
Circular genome map of *L. casei* KACC92338 (visualized using Proksee genome visualization tool). From the outer to inside: the first and second circles are forward and reverse CDS (coding sequences) annotated using Prokka, respectively, with tRNA, rRNA, tmRNA and Cas elements; the third circle is the GC content; the fourth circle depicts the GC skew (G-C)/(G + C), and the fifth circle represented the genome size (3,050,901 bp).

The sequenced whole genome of KACC92338 consists of a circular chromosome of length 3,050,901 bp (Genbank accession number SRR14601783), with an overall guanine-cytosine (GC) content of 47.96% (Figure 2), and devoid of plasmid DNA. The general features of the KACC92338 genome are presented in Table 3. The *L. casei* genomes are the second largest *Lactobacillus* genomes sequenced to date. Notably, the genome of KACC92338 was higher than *L. casei* ATCC 334 (Cai et al., 2009) and *L. casei* ATCC 393 (Toh et al., 2013), while GC content ratio was similar to the foresaid *L. casei* strains. In previous studies, a positive correlation was reported between the genome size and ubiquity, indicating strains with larger genomes adapt to a wider ecological niches (Cai et al., 2009; Guinane et al., 2016).

**Table 3:** Genomic information of *L. casei* KACC92338.

Attribute	Value
Genome size (bp)	3,050,901
GC content (%)	47.96
Contigs	63
N50 (bp)	169,757
L50	7
Plasmids	0
CDS	2,981
Total RNA’s	67 (57 tRNA +9 rRNA +1 tmRNA)
Total genes	3,048
Genes assigned to COGs	2,496

### 3.5 Species confirmation

The ANI-based taxonomy revealed that the genome of KACC92338 strain was most closely related with *Lacticaseibacillus casei* N87 (ANIb 96.55%; ANIm 96.92) and *L. casei* ATCC 393 (Tetra 0.99779).

**Figure 3:**
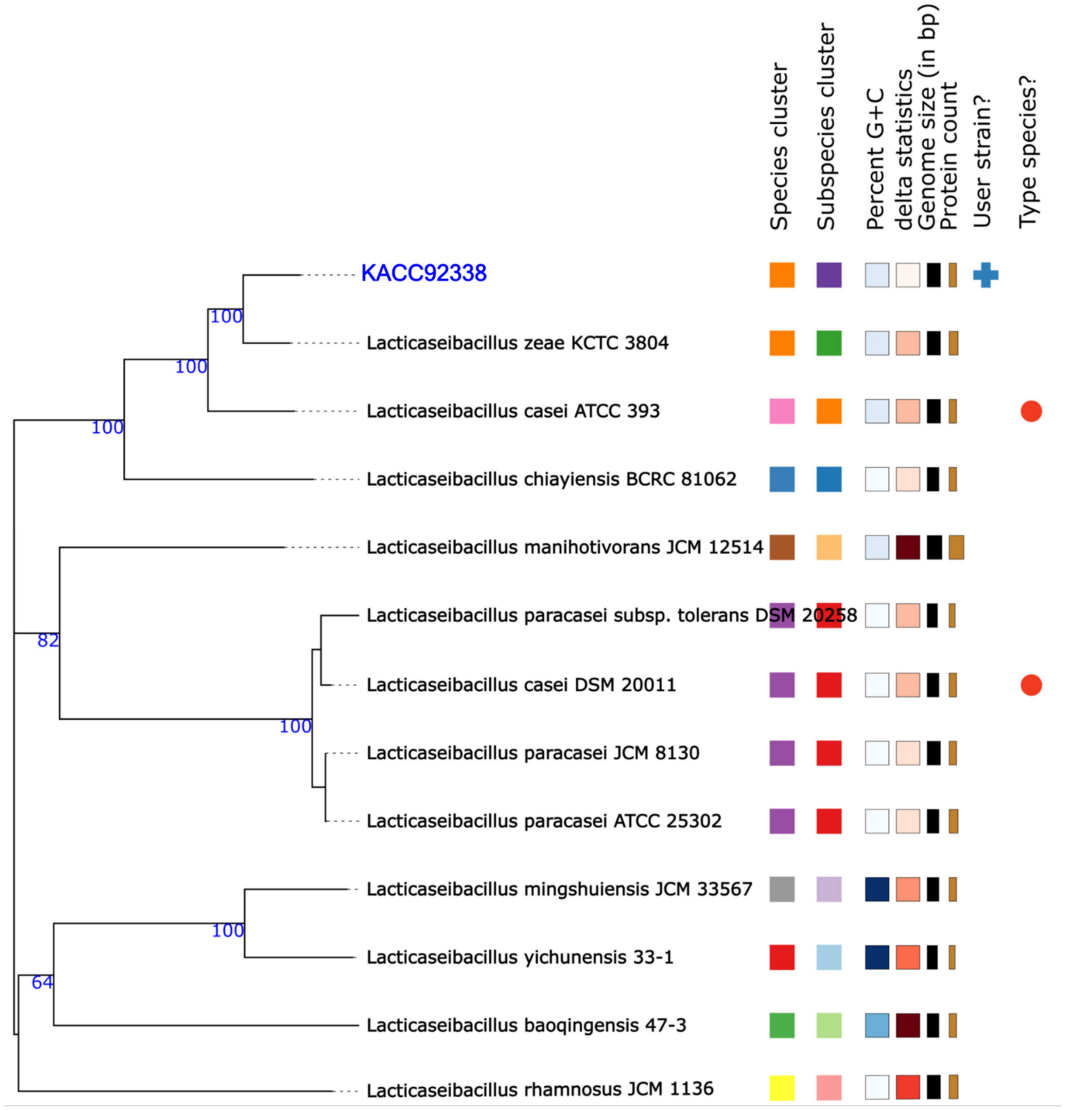
Phylogenetic tree based on TYGS result for the whole genome data set of *L. casei* KACC92338 with representative complete genomes of other *Lacticaseibacillus* strains. The tree was inferred with FASTME 2.1.6.1. from GBDP (genome BLAST distance phylogeny method) distances calculated from 16S rDNA gene sequences. The bootstrap support value before each node represents the confidence degree of each branch.

In addition, the genome-based taxonomic analysis conducted in TYGS web server indicated that KACC92338 strain showed similarity with other strains such as *Lacticaseibacillus casei* DSM 20011 and *L. casei* ATCC 393 (Figure 3). Therefore, the results of TYGS confirmed that KACC92338 indeed belongs to *L. casei* species.

### 3.6 Annotation

The genome annotation performed by Prokka revealed a total of 3,048 genes, with 2,981 protein-coding sequences (CDS), 57 tRNAs, 9 rRNAs, and 1 tmRNA (Table 3). Among the predicted CDS, 2135 genes (71.62%) were annotated with known functions, and the rest 846 genes (28.38%) were annotated as hypothetical proteins without any known functions. The gene coding for 57 tRNAs correspond to 20 natural amino acids: Arg (6 sequences); Gly, and Leu (5); Ala, Met, and Ser (4); Asn, Glu, Lys, and Thr (3); Asp., Gln, Ile, Phe, Pro, and Tyr (2); and Cys, His, Trp, and Val (1).

The analysis obtained from the RAST/SEED provided an overview of the coded biological features with a subsystem coverage of 42%, distributed in 232 subsystems (Table 4). The most represented subsystem features were involved in the metabolism of carbohydrates (25.43%), proteins (10.73%), amino acids and derivatives (7.38%), DNA (6.52%), and RNA (5.88%). Besides, 92 genes (5.31%) that participate in the production of a plethora of cofactors, vitamins, prosthetic groups and pigments were unveiled. The presence of genes encoding the biosynthesis of complex B vitamins (biotin, folate, pyridoxine, riboflavin, and thiamin) is a desirable trait of a probiotic, as humans are unable to synthesize B vitamins and is required as an external supplement. Further characterization of the biomolecules can prove advantageous in the food industry and can add to the nutritional value of food products.

**Table 4:** General overview of the RAST annotation and subsystem categories for the *L. casei* KACC92338 genome.

Description	Value	Percent
Cofactors, vitamins, prosthetic groups, pigments	92	5.31
Cell wall and capsule	95	5.48
Virulence, disease and defense	56	3.23
Potassium metabolism	4	0.23
Miscellaneous	23	1.33
Phages, prophages, transposable elements, plasmids	47	2.71
Membrane transport	65	3.75
Iron acquisition and metabolism	1	0.06
RNA metabolism	102	5.88
Nucleosides and Nucleotides	97	5.59
Protein metabolism	186	10.73
Cell division and cell cycle	47	2.71
Motility and chemotaxis	3	0.17
Regulation and cell signaling	46	2.65
DNA metabolism	113	6.52
Fatty acids, lipids, and isoprenoids	72	4.15
Dormancy and sporulation	5	0.29
Respiration	29	1.67
Stress response	57	3.29
Metabolism of aromatic compounds	4	0.23
Amino acids and derivatives	128	7.38
Sulfur metabolism	13	0.75
Phosphorus metabolism	28	1.61
Carbohydrates	441	25.43

### 3.7 Functional classification

**Figure 4:**
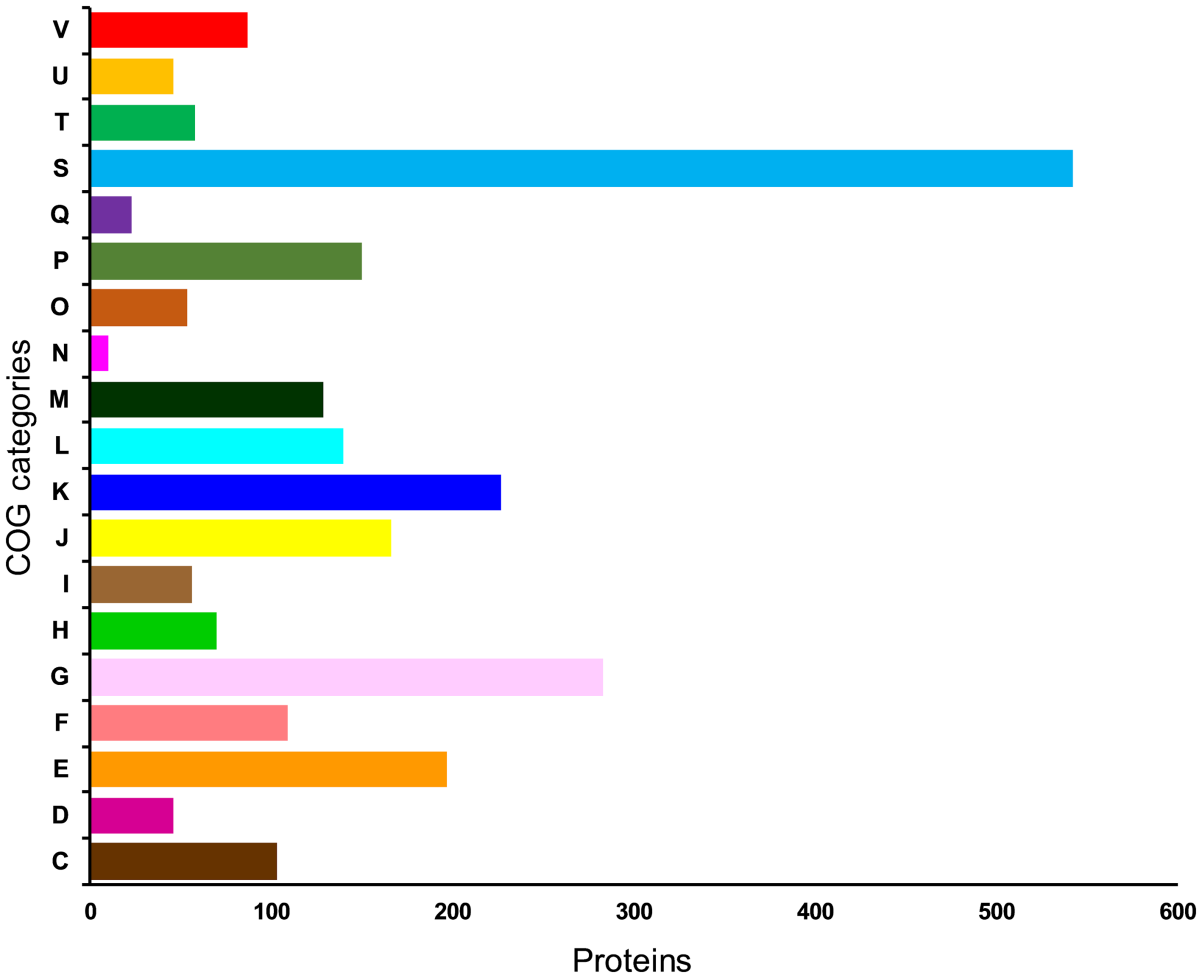
Distribution of genes across COG functional categories in the genome of *L. casei* KACC92338. In y-axis, C denotes energy production and conversion, D—cell cycle control, cell division, and chromosome partitioning; E—amino acid transport and metabolism; F—nucleotide transport and metabolism; G—carbohydrate transport and metabolism; H—coenzyme transport and metabolism; I—lipid transport and metabolism; J-translation, ribosomal structure and biogenesis; K—transcription; L—replication, recombination and repair; cell wall/membrane/envelope biogenesis; N—Cell motility; O—posttranslational modification, protein turnover, chaperones; P—inorganic ion transport and metabolism; Q—secondary metabolites biosynthesis, transport and catabolism; S—function unknown; T—signal transduction mechanisms; U—intracellular trafficking, secretion, and vesicular transport; and V—defense mechanisms.

To further understand the functional properties of KACC92338, the protein-encoding genes in the genome were annotated using COG and KEGG databases. Of the 2,981 CDS, 2496 (83.73%) genes were assigned to COG families comprising 19 functional categories by the EggNOG mapper, with the rest being functionally unknown. As shown in Figure 4, the most abundant COGs are carbohydrate transport and metabolism (G: 283); transcription (K: 227); amino acid transport and metabolism (E: 197); translation, ribosomal structure and biogenesis (J: 166); inorganic ion transport and metabolism (P: 150); replication, recombination and repair (L: 140); cell wall/membrane/envelope biogenesis (M: 129); nucleotide transport and metabolism (F: 109) and energy production and conversion (C: 103). Other than the above, defense mechanisms (V: 87); coenzyme transport and metabolism (H: 70); transduction mechanisms (T: 58); signal lipid transport and metabolism (I: 56); posttranslational modification, protein turnover, chaperones (O: 54); cell cycle control, cell division, chromosome partitioning (D: 46); intracellular trafficking, secretion, and vesicular transport (U: 46); secondary metabolites biosynthesis 23), and Cell motility (N: 10) were found to be enriched in KACC92338. The highest proportion of genes were under the function unknown (S: 542) category which contains several proteins related to phages, CRISPR, transport, and stress. The functional assignment was consistent with the previous studies (Wuyts et al., 2017; Fontana et al., 2018), highlighting the necessity for further enhancements in functional gene prediction. The higher number of genes involved in carbohydrate and amino acids metabolisms indicate our strains ability to utilize and degrade a large number of carbohydrate and proteins (Kim et al., 2022). The former attributes are mainly correlated to the large genome size similar to *L. plantarum* (Fontana et al., 2018).

Concerning the KEGG pathway database, approximately half of the CDSs (49.39%, 1,410 genes) were classified into 22 different functional categories (Table 5). Among them, the most abundant belong to carbohydrate metabolism (8.55%), protein families: genetic information processing (7.04%), protein families: signaling and cellular processes (5.95%), genetic information processing (5.71%), environmental information processing (4.76%), unclassified: metabolism (2.77%), amino acid metabolism (2.35%), and nucleotide metabolism (2.17%).

**Table 5:** KEGG orthology (KO) categories of identified protein-coding genes in the *L. casei* KACC92338 genome.

KO number	Functional category	Gene number	Proportion (%)
9101	Carbohydrate metabolism	244	8.55
9102	Energy metabolism	31	1.09
9103	Lipid metabolism	33	1.16
9104	Nucleotide metabolism	62	2.17
9105	Amino acid metabolism	67	2.35
9106	Metabolism of other amino acids	17	0.60
9107	Glycan biosynthesis and metabolism	33	1.16
9108	Metabolism of cofactors and vitamins	37	1.30
9109	Metabolism of terpenoids and polyketides	8	0.28
9110	Biosynthesis of other secondary metabolites	2	0.07
9111	Xenobiotics biodegradation and metabolism	3	0.11
9120	Genetic information processing	163	5.71
9130	Environmental information processing	136	4.76
9140	Cellular processes	20	0.70
9150	Organismal systems	5	0.18
9160	Human diseases	3	0.11
9181	Protein families: metabolism	51	1.79
9182	Protein families: genetic information processing	201	7.04
9183	Protein families: signaling and cellular processes	170	5.95
9191	Unclassified: metabolism	79	2.77
9192	Unclassified: genetic information processing	26	0.91
9193	Unclassified: signaling and cellular processes	19	0.67

These findings of the functional annotation analysis revealed that the KACC92338 strain has high metabolic capacity with abundant functional categories of genes responsible for carbohydrate and amino acid transport and metabolism, followed by genetic information processing and protein families: signaling and cellular processes, which indicates their importance in conserved cellular processes in this genome to enable the strain to adapt extreme environments and probiotic functions.

### 3.8 Probiotic attributes

The ability of probiotics to survive and establish themselves under harsh conditions (temperature, pH, bile, osmotic pressure, and oxidative stress) such as the gastrointestinal tract (GIT) and fermented food industries, and the ability of adhesion are critical parameters when choosing new probiotic strains (Hill et al., 2014; Evivie et al., 2017). Generally, lactobacilli strains have mechanisms to resist the environmental stress within the host to exert probiotic effects. *In silico* genome analysis of probiotic characteristics of *L. casei* KACC92338 unveiled several genes coding for temperature resistance, acid and bile tolerance, adhesion ability, oxidative stress, and immunomodulation, as listed in Table 6.

**Table 6:** Prediction of probiotic property-related functional genes in *L. casei* KACC92338 genome.

**Location**	**KEGG ID**	**Gene**	**Function**	Gene Nos.
Temperature				
*Heat stress*				
contig00007_10	K13993	*HSP20*	HSP20 family protein	2
contig00024_3	K04083	*hslO*	Molecular chaperone Hsp33	1
contig00002_11	K03686	*dnaJ*	Molecular chaperone DnaJ	1
contig00002_12	K04043	*dnaK*	HSPA9; molecular chaperone DnaK	1
contig00002_13	K03687	*grpE*	Molecular chaperone GrpE	1
contig00002_14	K03705	*hrcA*	Heat-inducible transcriptional repressor	1
contig00006_149	K03708	*ctsR*	Transcriptional regulator of stress and heat shock response	1
contig00001_204 (175)	K01419	*hslV*	ATP-dependent HslUV protease, peptidase subunit HslV [EC:3.4.25.2]	1
contig00001_205 (470)	K03667	*hslU*	ATP-dependent HslUV protease ATP-binding subunit HslU	1
contig00003_26 (94)	K04078	*groES*	HSPE1; chaperonin GroES	1
contig00003_27 (546)	K04077	*groEL*	HSPD1; chaperonin GroEL [EC:5.6.1.7]	1
contig00003_232 (718)	K04086	*clpL*	ATP-dependent Clp protease ATP-binding subunit ClpL	1
contig00001_137 (417)	K03544	*clpX*	CLPX; ATP-dependent Clp protease ATP-binding subunit ClpX	1
contig00001_152 (869)	K03695	*clpB*	ATP-dependent Clp protease ATP-binding subunit ClpB	1
contig00002_210 (700)	K03697	*clpE*	ATP-dependent Clp protease ATP-binding subunit ClpE	2
contig00006_148	K03696	*clpC*	ATP-dependent Clp protease ATP-binding subunit ClpC	1
contig00014_50	K01358	*clpP*	ATP-dependent Clp protease, protease subunit	1
contig00001_123 (363)	K07177	*lon*	Lon-like protease	1
Cold stress				
contig00001_86 (74)	K03704	cspA	Cold shock protein	1
contig00001_86	K03704	cspB	Cold shock protein	1
contig00008_13	K03704	cspC	Cold shock protein	1
Acid				
contig00002_37 (217)	K00655	plsC	1-acyl-sn-glycerol-3-phosphate acyltransferase [EC:2.3.1.51]	1
contig00001_162 (589)	K00873	pyk	Pyruvate kinase [EC:2.7.1.40]	1
contig00018_31	K02111	atpA	F-type H+/Na + −transporting ATPase subunit alpha [EC:7.1.2.2 7.2.2.1]	1
contig00018_27	K02108	atpB	F-type H + -transporting ATPase subunit a	1
contig00018_34	K02114	atpC	F-type H + -transporting ATPase subunit epsilon	1
contig00018_33	K02112	atpD	F-type H+/Na + −transporting ATPase subunit beta [EC:7.1.2.2 7.2.2.1]	1
contig00018_28	K02110	atpE	F-type H + -transporting ATPase subunit c	1
contig00018_29	K02109	atpF	F-type H + -transporting ATPase subunit b	1
contig00018_32	K02115	atpG	F-type H + -transporting ATPase subunit gamma	1
contig00018_30	K02113	atpH	F-type H + -transporting ATPase subunit delta	1
contig00009_96	K03316	nhaK	Sodium proton antiporter	1
contig00004_9	-	Asp2	Alkaline shock protein (Asp23) family	1
contig00004_10	-	Asp23	Alkaline shock protein (Asp23) family	1
Bile				
contig00001_213 (311)	K15986	ppaC	Manganese-dependent inorganic pyrophosphatase [EC:3.6.1.1]	1
contig00003_159 (394)	K00574	cfa	Cyclopropane-fatty-acyl-phospholipid synthase [EC:2.1.1.79]	1
contig00003_221 (373)	K01854	glf	UDP-galactopyranose mutase [EC:5.4.99.9]	1
contig00002_98 (447)	K01915	glnA	Glutamine synthetase [EC:6.3.1.2]	1
contig00001_12 (547)	K15580	oppA	Oligopeptide transport system substrate-binding protein	4
contig00003_208 (308)	K15581	oppB	Oligopeptide transport system permease protein	1
contig00003_209 (333)	K15582	oppC	Oligopeptide transport system permease protein	1
contig00003_210 (351)	K15583	oppD	Oligopeptide transport system ATP-binding protein	1
contig00003_211 (327)	K10823	oppF	Oligopeptide transport system ATP-binding protein	1
contig00001_113 (468)	K00382	lpd	Dihydrolipoyl dehydrogenase [EC:1.8.1.4]	1
contig00001_262 (635)	K07386	pepO	Putative endopeptidase [EC:3.4.24.-]	1
contig00001_50 (204)	K02986	rpsD	Small subunit ribosomal protein S4	1
contig00001_128 (85)	K02968	rpsT	Small subunit ribosomal protein S20	1
contig00001_129 (90)	K02956	rpsO	Small subunit ribosomal protein S15	1
contig00001_176 (438)	K02945	rpsA	Small subunit ribosomal protein S1	1
contig00001_302 (59)	K02970	rpsU	Small subunit ribosomal protein S21	1
contig00002_34 (263)	K02967	rpsB	Small subunit ribosomal protein S2	1
contig00002_49 (92)	K02959	rpsP	Small subunit ribosomal protein S16	1
contig00001_153 (64)	K02911	rpmF	Large subunit ribosomal protein L32	1
contig00002_45 (116)	K02884	rplS	Large subunit ribosomal protein L19	1
contig00002_67 (62)	K02902	rpmB	Large subunit ribosomal protein L28	1
contig00002_92 (97)	K02899	rpmA	Large subunit ribosomal protein L27	1
contig00002_94 (104)	K02888	rplU	Large subunit ribosomal protein L21	1
contig00002_142 (119)	K02887	rplT	Large subunit ribosomal protein L20	1
contig00002_143 (67)	K02916	rpmI	Large subunit ribosomal protein L35	1
contig00004_95 (152)	K02939	rplI	Large subunit ribosomal protein L9	1
Osmosis				
contig00004_146 (416)	K05847	opuA	Osmoprotectant transport system ATP-binding protein [EC:7.6.2.9]	1
contig00004_147 (210)	K05846	opuBD	Osmoprotectant transport system permease protein	1
contig00004_148 (310)	K05845	opuC	Osmoprotectant transport system substrate-binding protein	1
contig00004_149 (221)	K05846	opuBD	Osmoprotectant transport system permease protein	1
contig00011_53	K05845	choS	Periplasmic glycine betaine choline-binding (lipo)protein of an ABC-type transport system (osmoprotectant binding protein)	1
contig00003_121 (408)	K02000	proV	Glycine betaine/proline transport system ATP-binding protein [EC:7.6.2.9]	1
contig00003_122 (284)	K02001	proW	Glycine betaine/proline transport system permease protein	1
contig00003_123 (302)	K02002	proX	Glycine betaine/proline transport system substrate-binding protein	1
Adhesion				
contig00007_140	K00691	mapA	Maltose phosphorylase	1
contig00001_250 (156)	K03101	lspA	Lipoprotein signal peptidase II [EC:3.4.23.36]	1
contig00001_134 (397)	K02358	tuf	Elongation factor Tu	1
contig00003_128 (234)	K07284	srtA	Sortase A [EC:3.4.22.70]	1
contig00003_219 (305)	K19420	epsA	Protein tyrosine kinase modulator	1
contig00003_220 (249)	K00903	epsB	Protein-tyrosine kinase [EC:2.7.10.3]	1
contig00001_119 (1146)	K01958	pyc	Pyruvate carboxylase [EC:6.4.1.1]	1
contig00014_46	K01803	tpiA	Triosephosphate isomerase (TIM) [EC:5.3.1.1]	1
contig00014_48	K00134	gapA	Glyceraldehyde 3-phosphate dehydrogenase (phosphorylating) [EC:1.2.1.12]	1
contig00016_18	K12308	bgaC	Beta-galactosidase	1
contig00014_45	K01689	eno	Enolase [EC:4.2.1.11]	1
contig00008_1	K01810	pgi	Glucose-6-phosphate isomerase [EC:5.3.1.9]	1
Gut persistence				
contig00005_156	K02759	celC	PTS system, Lactose/Cellobiose specific IIA subunit	1
contig00003_197 (476)	K02761	celB	Cellobiose PTS system EIIC component	1
contig00003_200 (111)	K02759	celC	Cellobiose PTS system EIIA component	1
contig00003_17 (109)	K02760	celA	Cellobiose PTS system EIIB component	1
Oxidative stress				
contig00009_1	K11065	tpx	Thiol peroxidase	1
contig00016_53	K03671	trxA	Thioredoxin	2
contig00014_60	K00384	trxB	Thioredoxin reductase	1
contig00004_133 (397)	K03885	ndh	NADH dehydrogenase	1
contig00013_42	K05910	npr	NADH peroxidase	1
contig00004_30	K17869	nox	NADH oxidase	2
contig00011_12	K03781	katA	Catalase	1
contig00001_258 (77)	K06191	nrdH	Glutaredoxin	1
contig00003_80 (445)	K00383	GSR, gor	Glutathione reductase (NADPH) [EC:1.8.1.7]	1
contig00002_223 (585)	K00158	poxL	Pyruvate oxidase [EC:1.2.3.3]	1
contig00006_33	K03322	mntH	Manganese transport protein	3
contig00006_41	K19973	mntA	Manganese transport system ATP-binding protein [EC:7.2.2.5]	1
contig00006_43	K19976	mntB	Manganese transport system permease protein	1
contig00001_7 (279)	K07304	msrA	Peptide-methionine (S)-S-oxide reductase [EC:1.8.4.11]	1
contig00001_190	K07304	msrA	Peptide-methionine (S)-S-oxide reductase	1
contig00001_306	K07305	msrB	Peptide-methionine (R)-S-oxide reductase	1
contig00001_306 (148)	K07305	msrB	Peptide-methionine (R)-S-oxide reductase [EC:1.8.4.12]	1
contig00001_51 (161)	K08968	msrC	L-methionine (R)-S-oxide reductase [EC:1.8.4.14]	1
contig00006_138	K03386	ahpC	C-terminal domain of 1-Cys peroxiredoxin	1
Immunomodulation				
contig00019_32	K03367	dltA	D-alanine--poly(phosphoribitol) ligase subunit 1 [EC:6.1.1.13]	1
contig00019_33	K03739	dltB	Membrane protein involved in D-alanine export	1
contig00019_34	K14188	dltC	D-alanine--poly(phosphoribitol) ligase subunit 2 [EC:6.1.1.13]	1
contig00019_35	K03740	dltD	D-alanine transfer protein	1
Metal resistance				
contig00004_87 (618)	K01534	zntA	Zn2+/Cd2 + −exporting ATPase [EC:7.2.2.12 7.2.2.21]	1
contig00004_138 (620)	K01534	zntA	Zn2+/Cd2 + −exporting ATPase [EC:7.2.2.12 7.2.2.21]	1
contig00003_252 (151)	K02076	zurR	zur; Fur family transcriptional regulator, zinc uptake regulator	1
contig00011_90	K16264	czcD	Cobalt-zinc-cadmium efflux system protein	1
contig00015_43	K03284	corA	CorA-like Mg2+ transporter protein	1

Probiotic LAB capable of surviving in the GIT tend to contain several stress response genes which are beneficial to adapt to the intestinal environment and promote health effects (Evivie et al., 2017). The strain *L. casei* KACC92338 contains four universal stress proteins (*yugI, uspA, usp1, usp5*) induced under numerous stress conditions were present in the genome. For tolerance to higher temperatures, our strain harbored 20 genes coding for heat shock proteins in its genome. These included heat shock-related regulators (*hrcA, ctsR*), molecular chaperones (*dnaK, dnaJ, grpE, groEL, groES*, *hslO*), and protease-encoding genes (*hslU*, *hslV*, *lon*, *clpB*, *clpC*, *clpE*, *clpL*, *clpP, clpX*). These genes have been proven for upregulated expression in *Lactobacillus* strains to prevent intracellular protein aggregation and membrane stabilization during heat stress conditions (Kim et al., 2022; Li et al., 2023). In addition, three genes (*cspA, cspB, cspC*) encoding cold shock proteins for survival under low temperatures were also identified. The CSP family genes are synthesized in several *Lacticaseibacillus* strains (Hill et al., 2018; Kiousi et al., 2022; Liu et al., 2023) to overcome the deleterious effect under cold stress, and hence, KACC92338 May have a similar functional property. Function.

Concerning the acid tolerance mechanism, the KACC92338 genome possessed 11 genes encoding resistance at low pH levels. Out of them, the key proteins F_1_F_0_ ATP synthase (an atp operon comprising eight genes, *atpA, atpB, atpC, atpD, atpE, atpF, atpG, atpH*) and sodium proton antiporter (*nhaK*) play a crucial role in maintaining a stable intracellular pH in the bacterial cytoplasm for survival under acidic environment or acid stress (Kiousi et al., 2022; Li et al., 2023). Additionally, a *nhaK* gene coding for sodium-proton (Na+/H+) antiporters (maintain pH and Na + homeostasis) along with two alkaline shock protein genes (*Asp*2, *Asp*23) were present. All these genes suggest the KACC92338 strain’s ability to adapt under acidic and alkaline environments. Concerning resistance to bile salts, 29 genes namely inorganic pyrophosphatase (*ppaC,* maintain surface tension and keep membrane integrity), cyclopropane-fatty-acyl-phospholipid [CFP] synthase (*cfa,* enhance lipid synthesis), UDP-galactopyranose mutase (*glf*) glutamine synthetase (*glnA*), oligopeptide transporting proteins, and small and large subunit ribosomal proteins were identified. A similar bile-resistance mechanism was confirmed in *L. petauri* LZys1 through genome and phenotype analysis (Li et al., 2021). For enduring osmotic stress in the GIT, the KACC92338 genome contained eight genes (*opuA*, *opuC, opuBD, choS*, *proV*, *proW, proX*) that are responsible for uptake and accumulation of osmoprotectants such as glycine betaine, choline or proline. Strains of the former *L. casei* group employed as starter or nonstarter in food industries generally present high tolerance to heat and osmotic stress conditions (Kiousi et al., 2022).

The adhesion ability of probiotics to colonize the epithelial cells of the GIT can vary depending on the LAB species or strain, as their cell surface proteins play a significant role in interaction with the environment or host (Monteagudo-Mera et al., 2019). KACC92338 genome encodes 12 genes putatively coding for adhesion-related proteins, such as maltose phosphorylase (*mapA*), lipoprotein signal peptidase II (*lspA*), elongation factor Tu (*tuf*), sortase A (*srtA*), and enolase (*eno*), providing evidence of high adhesion ability. Besides, four genes (*celC, celB, and celA*) related to gut persistence were also present in the genome. These adhesions related genes can provide stability to the strain and prolong its antagonistic effect on unwanted gut microorganisms, aiding in effective colonization of the intestinal environment and inhibition of pathogens. Similar genes related to adhesion were earlier reported in *Lacticaseibacillus* strains (Kim et al., 2022; Kiousi et al., 2022; Rodrigo-Torres et al., 2022).

In recent years, there has been increasing attention to probiotic bacteria with oxidative stress tolerance and antioxidant properties for investigation in the treatment of various chronic human diseases (Kang et al., 2017; Vasconcelos et al., 2019). In this study, *L. casei* KACC92338 harbors 23 antioxidant functional genes related to oxidative stress, and out of them, 7 genes encode the complete thioredoxin (*tpx, trxA, trxB*) and NADH (*ndh, npr, nox*) antioxidant systems involved in ROS scavenging (Table 4). The thioredoxin system provides electrons to thiol-dependent peroxidases to catalyze the reduction of ROS and RNS at a fast reaction rate. The NADH oxidase cooperates with NADH peroxidase to eliminate hydrogen peroxide, enabling the degradation of ROS (Zhang et al., 2018). The presence of catalase (*katA*) gene can also significantly improve the antioxidant activity of our strain. These results support the findings of H_2_O_2_ tolerance, *in vitro* antioxidant and protective effect demonstrated by our strain in section 3.1 to 3.3. In addition, KACC92338 encoded genes for catalase (*katA*), pyruvate oxidase (*poxL*), and glutaredoxin (*nrdH*). Similar to the findings of *L. paracasei* SP5 (Kiousi et al., 2022), intracellular Mn*(II)* [manganese transport systems (*mntA*, *mntB*) and protein (*mntH*)] accumulation in KACC92338 can act as a functional replacement for superoxide dismutase. Moreover, the methionine sulfoxide reductase system (*msrA, msrB, msrC*) witnessed in our genome can catalyze the oxidized methionine residues formed by ROS and RNS in proteins. These results indicated that the strain KACC92338 might be a good probiotic candidate with potential antioxidant capacity that can support survival and damage repair under aerobic conditions.

Additionally, the KACC92338 genome is equipped with genes coding for immunomodulatory activities (*dlt A-D*). These results suggest that *L. casei* KACC92338 might resist multiple stress conditions and be consistent with the adaptability characteristics of the gastrointestinal tract.

### 3.9 Carbohydrate-active enzymes

Carbohydrate-active enzymes (CAZymes) are produced by the microbiome in the human intestinal gut and play a key role in the synthesis and degradation of complex polysaccharides and their derivatives (El Kaoutari et al., 2013). Using dbCAN3, the KACC92338 strain was found to harbor 201 genes classified under five CAZy classes (Supplementary Table S1). These numbers were relatively more abundant than those for *L. casei* FBL6 (Kim et al., 2022). The genes annotated by CAZy were dominated by the family of glycoside hydrolases (GHs, *n* = 170), followed by glycosyl transferases (GTs, *n* = 24), polysaccharide lyases (PLs, *n* = 4), carbohydrate-esterases (CEs, *n* = 2), and auxiliary activity (AA, *n* = 1). The genes of GHs were clustered in 53 different families, among which GH 13, GH 30, and GH 43 are dominant. GHs are key enzymes that hydrolyze the glycosidic bonds of carbohydrates and thereby release abundant energy to support various bacterial activities, indicating that the KACC92338 strain has the potential to utilize a wide range of complex carbohydrates (El Kaoutari et al., 2013). The second most abundant, GTs (clustered in 9 different families) catalyze the transfer of sugars from activated donor molecules to specific acceptors and are pivotal in forming surface structures that can be recognized by the host immune system (Mazmanian et al., 2008). The relatively high diversity of GH and GT in the genome can help the *L. casei* strain to utilize various sugars and promote immune stimulation and pathogen defense (Zhang et al., 2018). The ability to metabolize a large number of carbohydrates, including those that are not found in milk-based environments, supports the hypothesis that KACC92338 is capable of inhabiting in diverse ecological niches.

Moreover, a manual search in functional annotation disclosed few sugar transporter genes related to the phosphoenolpyruvate-dependent sugar phosphotransferase (PTS) system namely, beta-glucoside, cellobiose, fructose, galactitol, galactose amine, gluconate, lactose, mannitol, mannose, and sucrose indicating the strong carbon transporting ability of KACC92338, an important attribute for organisms that inhabit the gastrointestinal environment or can inhabit a variety of ecological niches. Interestingly, there were also genes encoding for enzymes such as acetaldehyde dehydrogenase, acetate kinase, alcohol dehydrogenase, D and L- lactate dehydrogenases, fructose-6-phosphate phosphoketolase, phosphate acetyltransferase, pyruvate formate-lyase, and xylulose-5-phosphate phosphoketolase. These enzymes play a crucial role in both homo- or hetero-fermentative pathways to produce acetate or lactate, which suggests that KACC92338’s inclusion in fermented foods could be an asset in the food industry.

### 3.10 Mobile genetic elements (MGE)

#### 3.10.1 Insertion sequences

Insertion sequences (IS) are small (<2.5 kb) segments of DNA with a simple organization, capable of inserting at multiple sites in a target molecule, and generally only encode genetic information required for transposition. IS elements have been documented in many species of LAB (Siguier et al., 2014). Fourteen insertion sequence (IS) elements from two different families (IS1202 and IS6) were predicted in the genome of *L. casei* KACC92338 with the set threshold *E*-value of 0.00001 (Supplementary Table S2). The IS1202 family contained 11 copies of ISLrh5 (*L. rhamnosus*) element, with only one copy exhibiting a zero *E*-value. However, all copies of ISLrh5 shared 70% amino acid similarity with ISPein1 (*Pediococcus inopinatus*). The IS6 family contained a single copy of each ISXne2 and elements. ISXne2 shared 83% amino acid similarity with ISYps1 (*L. reuteri*), and ISS1Z differs from ISS1S by 11 bp (1 amino acid).

#### 3.10.2 Island viewer

In the KACC92338 genome, Island Viewer 4 predicted five specific regions ranging in length from 9,060 to 57,794 bp, which are designated as genomic islands (GIs) (Supplementary Table S3). A total of 182 genes were assigned within these GIs, among which a relatively higher number encoding for hypothetical proteins. Additionally, genes related to transposases, integrases, bacteriocin, teichoic acid synthesis, proteins involved in DNA protection and modification, and enzymes related to carbohydrate metabolism have been found. These genes could play a significant role in enhancing the adaptability and competitiveness of the organism within the environmental niche (Ho Sui et al., 2009). Importantly, no genes related to virulence factors or drug-resistance mechanisms were annotated within the GIs.

#### 3.10.3 CRISPR−Cas

The analysis of CRISPR sequences using CRISPR-CasFinder uncovered eight CRISPR arrays in the *L. casei* KACC92338 genome. Among them three are located in contigs 1 and 3 with strong evidence level 4 (Table 7A). Additional CRISPR arrays detected were with evidence level 1 were disregarded as they are potentially invalid.

**Table 7:** Putative CRISPR-Cas sequences coded by CRISPR arrays **(A)** and Cas-related enzymes **(B)** in *L. casei* KACC92338.

**(A)**								
**Contigs No.**	**Start**	**End**	**Length (bp)**	**Orientation**	**Consensus repeat** **No. of CRISPRs with Same Repeat (Crisprdb)**	**Repeat Length**	**No. of Spacers**	**Evidence Level**
Contig 1_1	18,302	19,266	964	Reverse	ATTTCAATTCACGCAGTCACGTAGACTGCGAC	32	14	4
Contig 1_2	28,168	28,929	761	Forward	GTCGCAGTCCACGTGACTGCGTGAATTGAAAT	32	11	4
Contig 3_1	65,921	68,068	2,147	Reverse	GTCTCAGGTAGATGTCAGATCAATCAGTTCAAGAGC	36	32	4
Contig 3_2	77,409	77,563	154	Reverse	CATTGCGACCAAGGTCCTTACACGCAGACTCCTGCGCC GGCGAACGCGTTATGGA	55	1	1
Contig 4_1	115,613	115,756	143	Unknown	GAGGCCACTGGTTTTCTCTGCTGCTCTCT TAAACGCGTTCAC	42	1	1
Contig 7_1	116,016	116,121	105	Unknown	CAGAAGCCTGCGTGTAAGGACCTC	24	1	1
Contig 7_2	118,568	118,682	114	Unknown	GCTCTCCGAAACGCGCTCACAGGCCCAGAA	30	1	1
Contig 19_1	40,721	40,836	115	Forward	ATAACGCGCTCCCTGGCGCAGGAGCCTGC	29	1	1

**(B)**							
**Sequence ID**	**Cas-Type/Subtype**	**Gene Status**	**System**	**Type**	**Begin**	**End**	**Strand**
contig00001_22	cas4_TypeI-II	Accessory	CAS	CDS	19,630	20,286	+
contig00001_23	cas1_TypeIC	Mandatory	CAS-TypeIC	CDS	20,286	21,317	+
contig00001_24	cas2_TypeI-II-III	Accessory	CAS	CDS	21,326	21,616	+
contig00001_25	cas5c_TypeIC	Mandatory	CAS-TypeIC	CDS	21,908	22,636	+
contig00001_26	cas8c_TypeIC	Mandatory	CAS-TypeIC	CDS	22,633	24,522	+
contig00001_27	cas7c_TypeIC	Mandatory	CAS-TypeIC	CDS	24,512	25,402	+
contig00001_28	cas3_TypeI	Accessory	CAS	CDS	25,407	27,857	+
contig00003_64	cas9_TypeII	Mandatory	CAS-TypeIIU	CDS	60,141	63,836	+
contig00003_65	cas1_TypeII	Accessory	CAS-TypeIIU	CDS	64,035	64,940	+
contig00003_66	cas2_TypeI-II-III	Accessory	CAS	CDS	64,918	65,223	+
contig00003_67	csn2_TypeIIA	Mandatory	CAS-TypeIIA	CDS	65,220	65,900	+
contig00016_29	cas4_TypeI-II	Mandatory	CAS	CDS	35,973	38,363	+
contig00019_47	cas3_TypeI	Forbidden	CAS	CDS	47,939	49,285	−
contig00019_47	cas3_TypeI	Forbidden	CAS	CDS	47,939	49,285	−
contig00024_12	cas3_TypeI	Forbidden	CAS	CDS	12,306	15,833	−
contig00024_22	cas3_TypeI	Forbidden	CAS	CDS	24,082	25,590	−
contig00024_12	cas3_TypeI	Forbidden	CAS	CDS	12,306	15,833	−
contig00024_22	cas3_TypeI	Forbidden	CAS	CDS	24,082	25,590	−

Out of the total seven mandatory CRISPR-associated protein, four belonged to the Cas_TypeIC (cas1, cas5, cas8, cas7) in contig 1, two were from the Cas_TypeIIU (cas9), and Cas_Type IIA (csn2) systems in contig 3, and one from the CAS (cas4_TypeI-II) system alone in contig 16 (Table 7B). The presence of CRISPR-associated (Cas) proteins in our genome can provide a strong defense against invasive mobile genetic elements (conjugative plasmids, insertion sequences, and phages), along with restricting the dissemination of resistance genes (virulence, antibiotic) through horizontal gene transfer (Kim et al., 2022). Besides, the CRISPR functional system also confers stability to the lactic acid bacteria genomes in the dairy industry and has potential applications in gene editing for the improvement of their biotechnological applications (Song et al., 2012).

#### 3.10.4 Prophages

Prophages are a critical part of prokaryotic genomes and are widely distributed in several genera of LAB, including *Lacticaseibacillus* sp. (Pei et al., 2021; Rodrigo-Torres et al., 2022). Furthermore, prophages serve as a source of new genes added to the genome that provide various beneficial traits, such as the acquisition of antibiotic resistance genes, adaptation in new environmental niches, enhanced adhesion ability, or even turning the bacteria pathogenic (Pei et al., 2021).

A total of six regions (Table 8) were identified in the KACC92338 genome by the PHASTER webtool. These regions include two intact (regions 3 and 5), one questionable (region 2), and three incomplete (regions 1, 4 and 6) prophages. The phage regions mainly consist of hypothetical proteins and phage-related proteins from *Lactobacillus*, *Staphylococcus,* and *Bacillus*. The largest (35.9 kb) intact phage in region 3 (1–35,929 bp) showed a maximum (51) protein matching and resembled Lactob_PLE3_NC_031125(19), which is the most encountered prophage in *L. casei* strains (Xin et al., 2017). The next intact phage at region 5 (236–17,284 bp, 17 proteins; 17 kb) resembled Lactob_Lc_Nu_NC_007501(16). The questionable phage in region 2 (126909–145,470 bp, 19 proteins; 18.5 kb) corresponded to Staphy_SA7_NC_048658(2). Regarding the incomplete prophages, region 1 (153336–169,757 bp, 9 proteins; 16.4 kb), 4 (269–17,980 bp, 18 proteins; 17.7 kb) and 6 (1371–8,004 bp, 12 proteins, 6.6 kb) were consistent with Lactob_J_1_NC_022756(4), Lactob_BH1_NC_048737(17) and Lactob_phiAT3_NC_005893(5), respectively.

**Table 8:** Detection of prophage regions in *L. casei* KACC92338 genome using PHASTER tool.

	Region length (Kb)	Completeness	Score	Total tRNA	Phage proteins	Hypothetical proteins	Total proteins	Region position (bp)	Most common phage (Number of Matching proteins)	GC%	attL/attR sites	Integrase ORF Start-Stop
1	16.4	incomplete	20	0	8	1	9	153,336–169,757	PHAGE_Lactob_J_1_NC_022756(4)	43.70	+	164,356–165,525
2	18.5	questionable	80	0	11	8	19	126,909–145,470	PHAGE_Staphy_SA7_NC_048658(2)	45.38	+	144,322–145,470
3	35.9	intact	150	0	48	3	51	1–35,929	PHAGE_Lactob_PLE3_NC_031125(19)	46.34	+	34,561–35,745
4	17.7	incomplete	30	0	18	0	18	269–17,980	PHAGE_Lactob_BH1_NC_048737(17)	45.24	−	−
5	17	intact	120	0	17	0	17	236–17,284	PHAGE_Lactob_Lc_Nu_NC_007501(16)	44.11	−	−
6	6.6	incomplete	20	1	10	2	12	1,371–8,004	PHAGE_Lactob_phiAT3_NC_005893(5)	44.75	−	2,724–3,893

Integrases were identified in four [PP_01651 (region 1), PP_01809 (region 2), PP_02851 (region 3), and PP_03065 (region 6)] out of the six regions. Integrases are well-recognized diagnostic markers for mobile DNA elements (prophages, pathogenicity islands, and integrative plasmids) in bacterial genomes (Xin et al., 2017). The sequences of attachment sites (attL/attR) were homologous in each intact phage. No virulence or AMR genes have been found within the intact prophages.

### 3.11 Safety-associated genes

#### 3.11.1 Antibiotic resistance genes (ARG)

The *in-silico* prediction of ARG in the genome of KACC92338 using the CARD database under default settings (perfect/strict option) did not return any hits. However, changing the parameter to a less stringent criterion (perfect/strict/loose option), resulted in 211 loose hits (Supplementary Table S4) related to ARG associated with antibiotic target alteration (64), antibiotic target protection (13), antibiotic efflux (179), reduced permeability to antibiotic (1), antibiotic inactivation (16) and antibiotic target replacement (4). A genome map depicting the location of both ARG and MGE visualized in the Proksee server (Grant et al., 2023) (Supplementary Figure S1) showed that there is no co-localization between them, indicating there is no risks of transferability. These findings were already confirmed in the earlier section on MGE.

Furthermore, KACC92338 did not yield a significant match for any resistance genes associated with known antibiotics available in the Resfinder 4.1 database (90% threshold and 60% minimum length). The MobileElementFinder service could not find any mobile genetic elements in relation to antimicrobial resistance genes and virulence factors. It’s important to note that both the CARD and Resfinder databases mainly focus on ARG of pathogenic bacteria, therefore the ARG of non-pathogenic bacteria (eg. *Lactobacillus*) are usually not included.

Moreover, a search using BlastKOALA in the KACC92338 genome revealed genes related to resistance against tetracycline, macrolide, beta-lactam, and cationic antimicrobial peptides (Table 9). However, the presence of these genes does not warrant that KACC92338 surely expresses this resistance. Notably, despite the possession of macrolide and beta-lactamase resistance genes, the strain *L. plantarum* BCC 9546 was sensitive to these antibiotics. This May be related to various factors such as the gene expression level and substrate specificity of the expressed product (Chokesajjawatee et al., 2020). Earlier studies have shown that many LAB genera exhibit intrinsic resistance to tetracycline and macrolide due to ribosome protection, antibiotic efflux, and associated efflux pump formation. In agreement with these findings, the resistance genes in the KACC92338 code for transporters and efflux pumps, mainly related to non-specific antimicrobial resistance mechanisms. It’s also worth noting that our genome does not contain plasmids, making the transfer of antibiotic resistance genes by plasmid impossible. However, additional phenotypic testing in terms of clinical decision-making is necessary to clarify whether ARG encodes active proteins or plays different roles. These results can serve as an initial screening of genotypic ARG profiles indicating that *L. casei* KACC92338 is safe and unlikely to transfer any ARG to other commensals.

**Table 9:** List of antimicrobial resistance genes identified by BlastKOALA and their locations in the genome of *L. casei* KACC92338.

Resistance	Description	KEGG ID	Gene name	Gene Location
Tetracycline resistance	Transporters	K08168	tetB; MFS transporter, DHA2 family, metal-tetracycline-proton antiporter	contig00013_51
Macrolide resistance	Transporters	K19350	lsa; lincosamide and streptogramin A transport system ATP-binding/ permease protein	contig00009_102
		K08217	mef; MFS transporter, DHA3 family, macrolide efflux protein	contig00001_220
beta-Lactam resistance	Bla system [MD:M00627]	K17836	penP; beta-lactamase class A [EC:3.5.2.6]	contig00005_41
Tetracycline resistance	efflux pump Tet38 [MD:M00704]	K08168	tetB; MFS transporter, DHA2 family, metal-tetracycline-proton antiporter	contig00013_51
Cationic antimicrobial peptide (CAMP) resistance	dltABCD operon [MD:M00725]	K03367	dltA; D-alanine--poly(phosphoribitol) ligase subunit 1 [EC:6.1.1.13]	contig00019_35
		K03739	dltB; membrane protein involved in D-alanine export	contig00019_34
		K14188	dltC; D-alanine--poly(phosphoribitol) ligase subunit 2 [EC:6.1.1.13]	contig00019_33
		K03740	dltD; D-alanine transfer protein	contig00019_32
	Lysyl-phosphatidylglycerol (L-PG) synthase MprF [MD:M00726]	K14205	mprF, fmtC; phosphatidylglycerol lysyltransferase [EC:2.3.2.3]	contig00012_93

#### 3.11.2 Virulence factors

In KACC92338, no virulent genes were found using a BLASTn search in VirulenceFinder. However, a total of 22 hits were predicted through comparison with VFDB, which mainly included ABC transport proteins, stress survival, immune modulation, and adherence. It’s worth noting that these factors contributing to virulence in pathogens are also beneficial in probiotic strains as they help to increase their fitness for survival in the gut under several physiological stress conditions (Ho Sui et al., 2009).

#### 3.11.3 Hazardous metabolites

The search for genes related to undesirable metabolites using BlastKoala identified hemolysin (*hlyIII* – K11068) and D-lactate dehydrogenase (K03778) in the KACC92338 genome. Further analysis revealed that the toxin gene (*hly*III) has 100% sequence identity with a predicted membrane channel-forming protein *YqfA*, hemolysin III family. The widespread of the *hlyIII* gene in commercial probiotic *Lactobacillus* spp. (Zafar and Saier, 2020) imply the strain harboring the gene is not considered a safety concern.

The presence of D-lactate dehydrogenase gene in the genome implies the production of D-lactic acid, which is an essential component in the cell wall peptidoglycan of several gram-positive bacteria, including *L. plantarum*. However, caution is necessary on the consumption of foods containing LAB with D-lactic producing ability as it can lead to acidosis in patients and infants with short-bowel syndrome and carbohydrate malabsorption (Bianchetti et al., 2017).

An analysis of genes related to the enzymes involved in biogenic amines (BA) synthesis is another essential probiotic attribute related to consumer safety issues. Based on the KEGG database search, there were no genes related to BA synthesis in our genome, indicating that the strain is a BA-non-producer and deemed safe.

The results from the Pathogen Finder (Cosentino et al., 2013) indicated our strain is a non-human pathogen with zero matches against known pathogenic gene families and a very low probability (0.179) of being a human pathogen. In conclusion, the results on the genetical analyses related to safety aspects highlighted the absence of negative characteristics and confirms that *L. casei* KACC92338 is a safe strain and suitable for use as a probiotic and in different food industry applications.

### 3.12 Genome mining for bacteriocins

**Figure 5:**
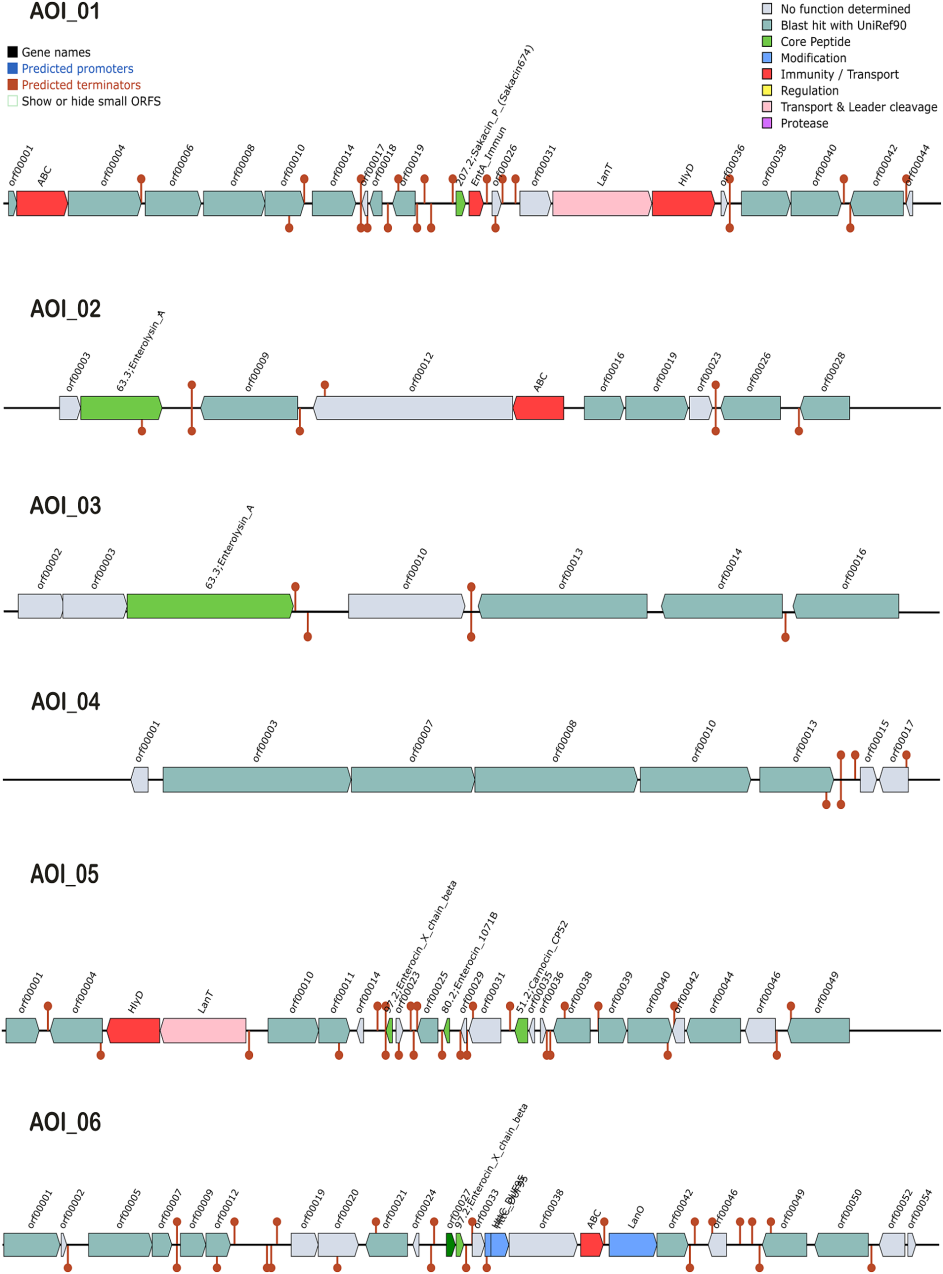
Graphical representation of biosynthetic gene clusters (BGCs) in *L. casei* KACC92338 genome predicted through the BAGEL4 webserver. Six contigs *viz.*, contigs 7.10 (AOI_01), 22.21 (AOI_02), 31.22 (AOI_03), 4.30 (AOI_04), 6.32 (AOI_05) and 12.6 (AOI_06) encoding genes potentially related to the biosynthesis of sakacin-P, Enterolysin_A, Enterolysin_A, sactipeptides, carnocin_CP52 and Enterocin X beta chain were identified. The BCGs are represented by arrows with different colors corresponding to the operons of different functions.

The antimicrobial activity of probiotics has a significant role in competing against GIT microbial pathogens by producing certain inhibitory substances, such as bacteriocins and lactic acid (Hernández-González et al., 2021). The biosynthetic gene clusters (BGC) in *L. casei* KACC92338 May have the ability to produce class II bacteriocins, sakacin-P, Enterolysin_A, sactipeptides and Enterocin X (Figure 5). These bacteriocins were predicted at six different locations (areas of interest (AOIs)) as follows: (i) contig 7.10 (AOI_01) (start at 145664, end at 165850), (ii) contig 22.21 (AOI_02) (start at 1, end at 11776), (iii) contig 31.22 (AOI_03) (start at 1, end at 6167), (iv) contig 4.30 (AOI_04) (start at 8053, end at 11947), (v) contig 6.32 (AOI_05) (start at 8740, end at 32412), and (vi) contig 12.6 (AOI_06) (start at 39605, end at 59758) suggesting the potential antimicrobial capacity of the strain.

The AOI_01 is comprised of 20,186 bp and contains 21 ORFs. The ORF 11 in this cluster codes for sakacin-P. Other genes in this cluster includes an ABC-type transporter, a putative immunity protein, a transport/processing ATP-binding protein (LanT), and an accessory factor for ABC-transporter PlnH (Hlyd). Protein sequence analysis of ORF11 by BLASTP showed high similarity to a class II leucocin A/sakacin P family bacteriocin (WP_070651009) from *Lacticaseibacillus*. A putative leucocin A/sakacin P was predicted previously in the genome of *L. casei* FBL6 (Kim et al., 2022). Sakacin P inhibits the growth of various gram-postive pathogens, especially *Listeria monocytogenes*, a common and persistent pathogen in the food industry (Katla et al., 2001).

The clusters AOI_02 (11,775 bp, 10 ORFs) and AOI_03 (6,166 bp, 7 ORFs) produce Enterolysin_A (Class III bacteriocin), a heat-resistant, cell-wall-degrading bacteriocin broadly dispensed among enterococcal strains. The bacteriocin was initially reported from *Enterococcus faecalis* LMG2333 (Nilsen et al., 2003) and has been found to be active against Gram-positive bacteria such as enterococci, pediococci, and lactococci. The AOI_02 includes a bacteriocin gene (ORF 2) and an ABC transporter (ORF 5), while AOI_03 contains only one bacteriocin gene (ORF3). The BLASTP analysis of enterolysin_A from AOI_02 correspond to N-acetylmuramoyl-L-alanine amidase (WP_093997694.1) of *Lacticaseibacillus*, while enterolysin_A from AOI_03 showed specific hits with lysozyme (WP_306402816.1) and N-acetylmuramoyl-L-alanine amidase (OFR73919.1) from *Lactobacillus* sp. HMSC061B07. N-acetylmuramoyl-L-alanine amidase destroys microbial cell walls leading to cell rupture and death.

The AOI_4 (3,894 bp, 8 ORFs) codes for the bacteriocin of sactipeptides (ribosomally synthesized and post-translationally modified peptides), while AOI_5 (23,672 bp, 23 ORFs) encodes carnocin_CP52 immunity protein (class II bacteriocin, ORF 14) that contain five genes including a bacteriocin secretion accessory protein (Hlyd), a peptide cleavage/export ABC transporter (LanT) and three predicted core peptides, including Enterocin X beta chain (ORF 8), Enterocin (ORF 11), and carnocin_CP52. Identification by BLASTp showed the gene sequence of Enterocin X beta chain showed 100% identity to a bacteriocin leader domain-containing protein reported in many strains of *Lacticaseibacillus zeae*, while Enterocin showed hits with lactococcin G-beta/enterocin 1071B family bacteriocin from *Weissella cibaria* and carnocin_CP52 showed 100% QC for bacteriocin immunity protein from several *Lacticaseibacillus* strains.

The AOI_6 (20,153 bp, 24 ORFs) encodes Enterocin X beta chain (Class IIc bacteriocin, circular peptide) which includes two class II bacteriocins (ORF 11, ORF 12), two uncharacterized proteins (ORF 14, ORF 15), ABC (ORF 17), and LanO (ORF 18). Based on BLASTp results, the bacteriocin at ORF11 was identified as a ComC/BlpC family leader-containing pheromone/ bacteriocin (WP_010492210.1) from *Lacticaseibacillus*, and ORF12 coding Enterocin X beta chain was found as a bacteriocin leader domain-containing protein reported in many strains of *L. zeae*.

The analysis concludes the ability of our strain to produce class II bacteriocins, that can contribute to the inhibitory effect against pathogens. The information presented here about the bacteriocins produced by this strain relies on the information available in the bacteriocin database and search programs employed. Further *in vitro* studies are necessary to confirm the exact bacteriocins or any additional proteins produced by the strain and under the conditions which they are produced.

## 4 Conclusion

The current study highlights the antioxidant property, whole genome sequence and *in silico* mining to retrieve genes related to probiotic characteristics, bacteriocin production, and genome stability of the *L. casei* KACC92338 strain. To our knowledge, this is the first whole-genome sequence of *L. casei* isolated from Korean raw milk and the first report on higher antioxidant activity by *L. casei* strain than reported earlier. The ability of tolerance towards H_2_O_2_, with potential *in vitro* scavenging activities and cellular protective effects on yeast cells indicated that *L. casei* KACC92338 possess good antioxidant capacity and the potential to reduce cellular damage caused by excessive oxidative stress in the host. *In silico* analysis of this strain showed various probiotic-associated genes that confer tolerance towards stress conditions of high and low temperatures, acid, bile, and oxidative stress, as well as abilities for adhesion, carbohydrate metabolism, and bacteriocin production. Moreover, safety assessments studies showed the absence of acquired antibiotic resistance genes, virulence factors, pathogenicity, or plasmids. The presence of prophage regions, CRISPR-Cas system, and insertion sequences further confirm genome stability. Overall, the results of genotypic validation serves as only the first step in safety assessment of *L. casei* KACC92338 and provides a direction for subsequent application. However, further confirmation through *in vivo* assessments are necessary to guarantee the safety and overall industrial stability of the strain.

## Data Availability

The datasets presented in this study can be found in online repositories. The names of the repository/repositories and accession number(s) can be found below: https://www.ncbi.nlm.nih.gov/search/all/?term=PRJNA731289.

## Footnotes

^1^https://usegalaxy.org/

^2^http://www.usadellab.org/cms/?page=trimmomatic

^3^http://bagel4.molgenrug.nl/

## Supplementary material

SUPPLEMENTARY FIGURE S1 Antibiotic resistance genes (ARG) and Mobile genetic elements (MGE) in *L. casei* KACC92338 genome (visualized using Proksee genome visualization tool).
