# Supplementary figures and images for: Whole genome sequencing of Lacticaseibacillus casei KACC92338 strain with strong antioxidant activity, reveals genes and gene clusters of probiotic and antimicrobial potential
Source: Front Microbiol. 2024 Sep 26;15:1458221. doi: 10.3389/fmicb.2024.1458221 (PMC11464305; doi:10.3389/fmicb.2024.1458221)

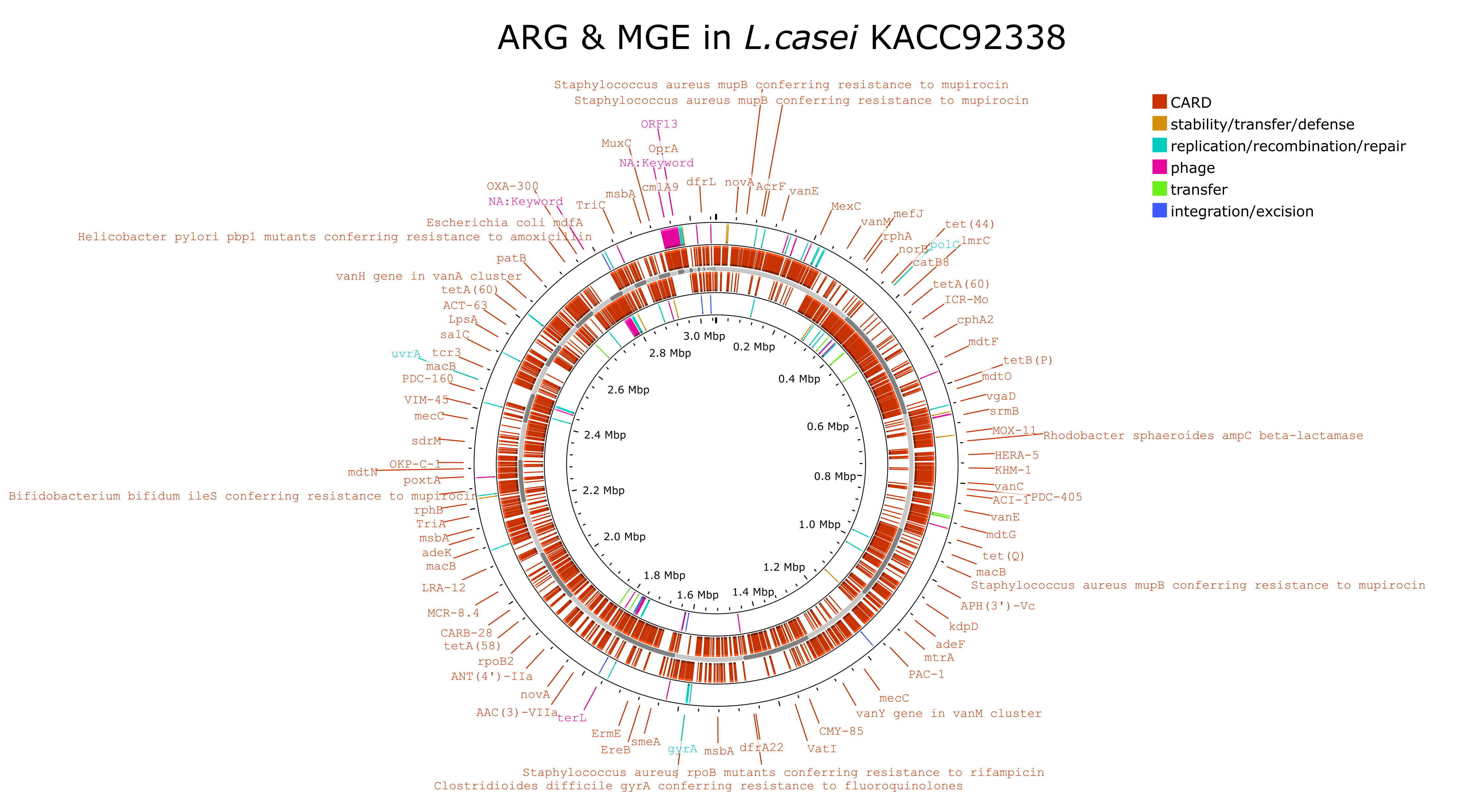

Supplement: SUPPLEMENTARY FIGURE S1 — Antibiotic resistance genes (ARG) and Mobile genetic elements (MGE) in L. casei KACC92338 genome (visualized using Proksee genome visualization tool). [file Image_1.TIFF]
